# Clinical and microbiological analyses of colistin-resistant strains among carbapenem-resistant *Enterobacter cloacae* complex clinical isolates

**DOI:** 10.1128/spectrum.01604-24

**Published:** 2024-12-31

**Authors:** Jinyoung Yang, Jin Yang Baek, Jae-Hoon Ko, Kyungmin Huh, Sun Young Cho, Doo Ryeon Chung, Kyong Ran Peck, Hee Jae Huh, Kwan Soo Ko, Cheol-In Kang

**Affiliations:** 1 Division of Infectious Diseases, Department of Medicine, Samsung Medical Center, Sungkyunkwan University School of Medicine, Seoul, South Korea; 2 Asia Pacific Foundation for Infectious Diseases (APFID), Seoul, South Korea; 3 Department of Laboratory Medicine and Genetics, Samsung Medical Center, Sungkyunkwan University School of Medicine, Seoul, South Korea; 4 Department of Microbiology, Sungkyunkwan University School of Medicine, Suwon, South Korea; Tainan Hospital, Ministry of Health and Welfare, Tainan, Taiwan

**Keywords:** *Enterobacter cloacae *complex, carbapenem, colistin, antibiotic resistance, molecular epidemiology

## Abstract

Carbapenem-resistant *Enterobacter cloacae* complex (CR-ECC), which is rapidly increasing as the cause of nosocomial infections, has limited treatment options. The aim of this study is to investigate the microbiological and clinical traits and molecular epidemiology of isolates of CR-ECC and provide guidance for antibiotic selection in clinical practice. Clinical CR-ECC isolates (ertapenem MIC ≥ 2 mg/L) were collected from 2021 to 2022. Species identification was performed using *hsp60* gene analysis, and antibiotic susceptibility tests were conducted by broth microdilution. The clinical characteristics of patients with CR-ECC isolates were retrospectively analyzed. Among the 108 CR-ECC isolates, 25 (23.2%) were non-susceptible to colistin, with colistin susceptibility being higher in *Enterobacter hormaechei* compared to non-*E*. *hormaechei* isolates (*P* < 0.0001). Of the 108 CR-ECC isolates, 9 (8.3%) produced carbapenemases, and only 6 of the 22 colistin-resistant CR-ECC isolates (27.3%) harbored the *mcr* gene. A total of 73 sequence types (STs), including 28 newly identified STs, were detected, demonstrating significant clonal diversity. The most common ST was ST74, known for its high prevalence and association with carbapenem resistance, with 77.8% identified as *E. hormaechei* subsp. *hoffmannii. E. hormaechei* was more common in the colistin-susceptible group than in the non-susceptible group (88.0% vs 37.5%, *P* < 0.0001), and *E. hormaechei* was the only protective factor for colistin resistance (HR 0.089, CI 0.030–0.261, *P* < 0.001). Although colistin resistance of CR-ECC is high, colistin could be administered safely to *E. hormaechei*. It is imperative to maintain ongoing surveillance and to further research on CR-ECC.

**IMPORTANCE:**

Although new antibiotics are being developed, there are still limited options for treating carbapenem-resistant *Enterobacter cloacae* complex (CR-ECC) in regions where their use is restricted. The resistance level to one of these options, colistin, was investigated using bacteria isolated from clinical samples. In clinical practice, colistin is frequently administered empirically without susceptibility testing. However, this study suggests that colistin can be safely administered to certain species such as *Enterobacter hormaechei* within the CR-ECC.

## INTRODUCTION

Carbapenem is a common treatment for severe infection with multidrug-resistant Gram-negative bacteria (GNB). Nonetheless, carbapenem-resistant bacteria have also increased rapidly due to the increase in its usage (1). This limits the choice of antibiotics available for the treatment of carbapenem-resistant Enterobacteriaceae (CRE) infections, resulting in poor prognosis and high mortality (2). Although colistin is used as the last-line treatment for CRE infection (3, 4), Enterobacteriaceae resistant to colistin are also rapidly increasing (5).

In South Korea, 30,548 cases of CRE infection were reported in 2022 according to the Korea Disease Control and Prevention Agency (KDCA) (6). *Enterobacter cloacae* ranks third in frequency among CREs isolated in South Korea over the past 5 years (7) and is emerging as an important cause of nosocomial infections, such as pneumonia, urinary tract infections, and bacteremia (8, 9). According to a previous study, the colistin resistance rate of Enterobacteriaceae was 0.67%, but the resistance rate of *E. cloacae* was 4.2%, which is higher than that of *Escherichia coli* or *Klebsiella pneumoniae* (10). Additionally, the *E. cloacae* complex (ECC) is divided into 13 genetic clusters through *heat shock protein 60 (hsp60*) gene sequencing (11), and there are studies that indicate differences in antibiotic susceptibility depending on these clusters (12, 13).

In this study, we performed *hsp60* gene analysis to identify carbapenem-resistant *E. cloacae* complex (CR-ECC) clinical isolates and the antibiotic susceptibility test (AST) for various antibiotics, including colistin, using the broth microdilution (BMD) method. Additional molecular analysis of the isolates was performed, and the clinical characteristics of the patients were analyzed. Through these efforts, we aim to investigate the characteristics and molecular epidemiology of CR-ECC and, in particular, the characteristics of clinical isolates showing colistin resistance.

## MATERIALS AND METHODS

### Selection of the CR-ECC isolates and analysis of clinical characteristics of patients

CR-ECC isolates were screened from CRE collection (ertapenem minimum inhibitory concentration, MIC ≥ 2 mg/L) acquired from January 2021 to December 2022 in Samsung Medical Center, a 1,989-bed tertiary hospital. Strains were screened from blood, urine, respiratory specimens, peritoneal fluid, pleural fluid, skin swab, feces, and rectal swabs. Primary identification of bacteria was through classification according to the results of the VITEK MS system (bioMerieux, Marcy-1Etoile, France), and secondary identification of specific species was through *hsp60* gene analysis. Our center used VITEK2 (bioMerieux, Marcy-1Etoile, France) for antibiotic susceptibility testing.

Electronic medical records were retrospectively reviewed for patients from whom CR-ECC was isolated to analyze clinical characteristics. Data collected through medical records were age, sex, Charlson comorbidity score, underlying conditions, true infection or colonization, type of infection, treatment success, hospital days, drainage catheter, location of bacterial detection, history of carbapenem or colistin administration, multidrug-resistant organism (MDRO) detection, hospitalization within 3 months, and hemodialysis. Patients were divided into two groups according to colistin susceptibility and also according to true infection or colonization, and baseline characteristics were compared. The criteria for determining true infection were as follows: first, cases where bacteria were isolated from blood samples were considered as confirmed infections, excluding those identified through rectal swabs. Additionally, clinical occurrences or worsening indicative of infection, such as fever, elevation in inflammatory markers, or worsening of symptoms, both before and after the detection of the bacteria, were included in the true infection. All other cases were classified as colonizers.

### Species identification by *hsp60* gene analysis

Species identification was performed by *hsp60* gene analysis according to a previous study (11), and the gene fragments were amplified and sequenced using primer set Hsp60-F/Hsp60-R. The obtained sequences of 264 bp were compared with the sequences of reference strains of 11 species or subspecies within the ECC, which were retrieved from the GenBank database (Table S1). The similarity of partial *hsp60* sequences between reference strains and clinical isolates of subspecies of *Enterobacter hormaechei* is shown in Table S2. *Hsp60* sequences were aligned with the ClustalW multisequence alignment program, and phylogenic trees were constructed using the neighbor-joining method and the MEGA 11.0 program package and iTOL software.

Genotypes were determined using the Oxford multilocus sequence typing (MLST) scheme (14). Seven housekeeping genes (*dnaA, fusA, gyrB, leuS, pyrG, rplB*, and *rpoB*) were amplified using primer sets, and new alleles were submitted to the *E. cloacae* typing database (https://pubmist.org/organisms/enterobacter-cloacae) and were assigned new numerical identifiers. A minimum spanning tree was constructed using phyloviz version 2.0a(15) based on the determined allelic profiles of sequence types (STs). The relatedness between STs was analyzed by eBURST (https://www.phyloviz.net/goeburst/)

### Antibiotic susceptibility test and detection of antibiotic resistance genes

For antibiotic susceptibility testing, 15 agents were included based on their common usage for treating carbapenem-resistant Gram-negative bacterial infections (16, 17): colistin (CST), amikacin (AMK), gentamicin (GEN), imipenem (IPM), meropenem (MEM), ertapenem (ETP), levofloxacin (LVX), ciprofloxacin (CIP), fosfomycin (FOF), cefepime (FEP), cefotaxime (CTX), aztreonam (ATM), tigecycline (TGC), ceftolozane/tazobactam (C/T), and piperacillin/tazobactam (TZP). The MICs were determined using the BMD and agar dilution method for fosfomycin, and the susceptibility breakpoints were interpreted in accordance with the Clinical and Laboratory Standards Institute (CLSI) guideline (18), except for that of tigecycline. For tigecycline, FDA-identified interpretive criteria for Enterobacteriaceae were used: susceptible (MIC, ≤2 mg/L), intermediate (MIC, 4 mg/L), and resistant (MIC, ≥8 mg/L) (19). *Escherichia coli* ATCC 25922 and *Pseudomonas aeruginosa* ATCC 27853 were used as control strains.

For carbapenem-resistant isolates, metallo-β-lactamase, OXA-48-like carbapenemases, and *Klebsiella pneumoniae* carbapenemase (KPC) genes were screened by PCR and sequencing (5, 20–23). For colistin-resistant isolates, *mcr* genes (*mcr*-1–10) were screened by single/multiplex PCR (24–27). The presence of target genes was confirmed by Sanger sequencing of PCR products.

### Statistical analysis

For comparative analysis of baseline characteristics, chi-square or Fisher’s exact tests were used for categorical variables. Logistic regression was used to analyze risk factors for colistin-resistant CR-ECC. All *P* values were calculated with two-sided tests, and those <0.05 were considered statistically significant. IBM SPSS Statistics version 28 (IBM, Armonk, NY, USA) and GraphPad Prism version 10.0 (GraphPad Software, San Diego, CA, USA) were used for all statistical analyses.

## RESULTS

### Species identification and antibiotic susceptibility tests of CR-ECC isolates

Among all 108 CR-ECC isolates, the most frequently identified species using *hsp60* was *E. hormaechei* subsp. *xiangfangensis* (*n* = 38, 35%), followed by *E. hormaechei* subsp. *steigerwaltii* (*n* = 32, 30%), *E. hormaechei* subsp. *hoffmannii* (*n* = 13, 12%), *Enterobacter ludwigii* (*n* = 7, 6%), *E. hormaechei* subsp. *hormaechei* (*n* = 6, 5%), *Enterobacter roggenkampii* (*n* = 6, 5%), *Enterobacter asburiae* (*n* = 6, 6%), *Enterobacter kobei* (*n* = 5, 5%), and *E. cloacae* subsp. *cloacae* (*n* = 1, 1%).

The isolated CR-ECC strains were tested for susceptibility to 15 antibiotics using the BMD method (Table 1). Among them, 25 strains (23.2%) showed non-susceptibility to colistin, 13 (12.0%) were resistant to imipenem, and 10 (9.3%) were resistant to meropenem. In addition, 58 (53.7%) were susceptible to piperacillin/tazobactam, while 31 (28.7%) were susceptible to cefepime, 25 (23.2%) were standard-dose dependent (SDD), and 52 (48.1%) were resistant to cefepime, showing a higher rate of resistance to cefepime than to piperacillin/tazobactam. Fluoroquinolone susceptibility was around 53%–54%, but amikacin susceptibility was relatively high (96.3%), as were susceptibility to tigecycline (71.3%) and fosfomycin (79.6%).

**TABLE 1:** Antibiotic susceptibility test results using broth microdilution method in carbapenem-resistant *Enterobacter cloacae* complex isolates

Antimicrobial agent	*N* (%)		
	Susceptible	Intermediate	Resistant
Colistin	83 (76.8)	3 (2.8)	22 (20.4)
Tigecycline	77 (71.3)	25 (23.1)	6 (5.6)
Ceftolozane/tazobactam	4 (3.7)	4 (3.7)	100 (92.6)
Piperacillin/tazobactam	58 (53.7)	10 (9.3)	40 (37.0)
Amikacin	104 (96.3)	3 (2.8)	1 (0.9)
Gentamicin	98 (90.7)	0	10 (9.3)
Imipenem	92 (85.2)	3 (2.8)	13 (12.0)
Meropenem	96 (88.9)	2 (1.8)	10 (9.3)
Ertapenem	0	0	108 (100)
Levofloxacin	59 (54.6)	7 (6.5)	42 (38.9)
Ciprofloxacin	58 (53.7)	4 (3.7)	46 (42.6)
Cefepime	31 (28.7)	25 (23.2)	52 (48.1)
Cefotaxime	1 (0.9)	0	107 (99.1)
Aztreonam	6 (5.5)	3 (2.8)	99 (91.7)
Fosfomycin	86 (79.6)	14 (13.0)	8 (7.4)

Nine (8.3%) CR-ECC isolates produced carbapenemase, including six NDM-1, two NDM-5, one IMP-1, and one KPC-2, of which one isolate was co-harboring NDM-1 and KPC-2. Two *E. ludwigii* and one *E. hormaechei* subsp. *hoffmannii* also had the *mcr-9* gene. All except one isolate were susceptible to colistin and amikacin and showed moderate susceptibility to tigecycline and fosfomycin, but only two isolates were sensitive to fluoroquinolone (Table S3).

### Comparison of antibiotic susceptibility tests between *E. hormaechei* and non-*E*. *hormaechei* isolates

When comparing the antibiotic susceptibility between *E. hormaechei* and non-*E*. *hormaechei* (Table 2), *E. hormaechei* isolates had a higher susceptibility rate for colistin (89.2% vs 36%, *P* < 0.0001), imipenem (91.6% vs 64%, *P* = 0.0019), meropenem (92.8% vs 76%, *P* = 0.0298), and fosfomycin (85.5% vs 60%, *P* = 0.0097) than non-*E*. *hormaechei* isolates. Non-*E*. *hormaechei* showed greater susceptibility than *E. hormaechei* isolates to tigecycline (66.3% vs 88%, *P* = 0.0437) and cefepime (19.3% vs 60%, *P* = 0.0002), and there was no difference between piperacillin/tazobactam (51.8% vs 60%, *P* = 0.5018) and amikacin (96.4% vs 96%, *P* > 0.9999). For cefepime, 25 cases of SDD (21 for *E. hormaechei* and 4 for non-*E*. *hormaechei*) were included, but there was no difference even if they were considered as susceptible (44.6% vs 76%, *P* = 0.0065). In the antimicrobial susceptibility results obtained by the BMD method, all strains pre-identified as *E. hormaechei* by VITEK MS, a method commonly used in clinical settings, were found to be susceptible to colistin.

**TABLE 2:** Comparison of the antimicrobial resistance rate between carbapenem-resistant *E. hormaechei* (*n* = 83) and carbapenem-resistant non-*E*. *hormaechei* (*n* = 25)

Species (No. of isolate)	CST	TGC	C/T	TZP	AMK	GEN	IPM	MEM	ETP	LVX	CIP	FEP	CTX	ATM	FOF
*E. hormaechei*															
subsp. *xiangfangensis*	0	7.9	92.1	50.0	2.6	10.5	5.0	5.0	100	39.5	44.7	50.0	100	89.5	0
subsp. *steigerwaltii*	18.8	3.1	100	31.3	0	6.3	3.1	3.1	100	25.0	31.3	62.5	100	93.8	6.3
subsp. *hoffmannii* (13)	7.7	15.4	84.6	38.5	0	15.4	23.1	23.1	100	69.2	69.2	54.0	100	92.3	7.7
Non-*E. hormaechei*															
*E. ludwigii* (7)	28.6	0	100	42.9	14.3	14.3	57.0	57.0	100	71.4	71.4	57.0	100	71.4	14.0
*E. roggenkampii* (6)	50	0	83.3	33.0	0	17.0	17.0	0	100	50.0	50.0	33.3	100	100	33.3
*E. asburiae* (6)	83	0	66.7	0	0	0	17.0	0	100	17.0	17.0	0	100	100	17.0
*E. kobei* (5)	60	0	100	20.0	0	0	0	0	100	20.0	20.0	0	100	100	20.0
*E. cloacae* subsp. *cloacae* (1)	100	0	100	0	0	0	0	0	100	0	0	0	100	100	0

Comparing the antibiotic susceptibility of subspecies among *E. hormaechei*, the most isolated *E. hormaechei* subsp. *xiangfangensis* had no colistin resistance and had high susceptibility to imipenem and meropenem. *E. hormaechei* subsp. *steigerwaltii* was the species most resistant to cefepime, and *E. hormaechei* subsp. *xiangfangensis* was the most resistant to piperacillin/tazobactam.

### Multilocus sequence typing and clonal diversity of CR-ECC isolates

**Fig 1:**
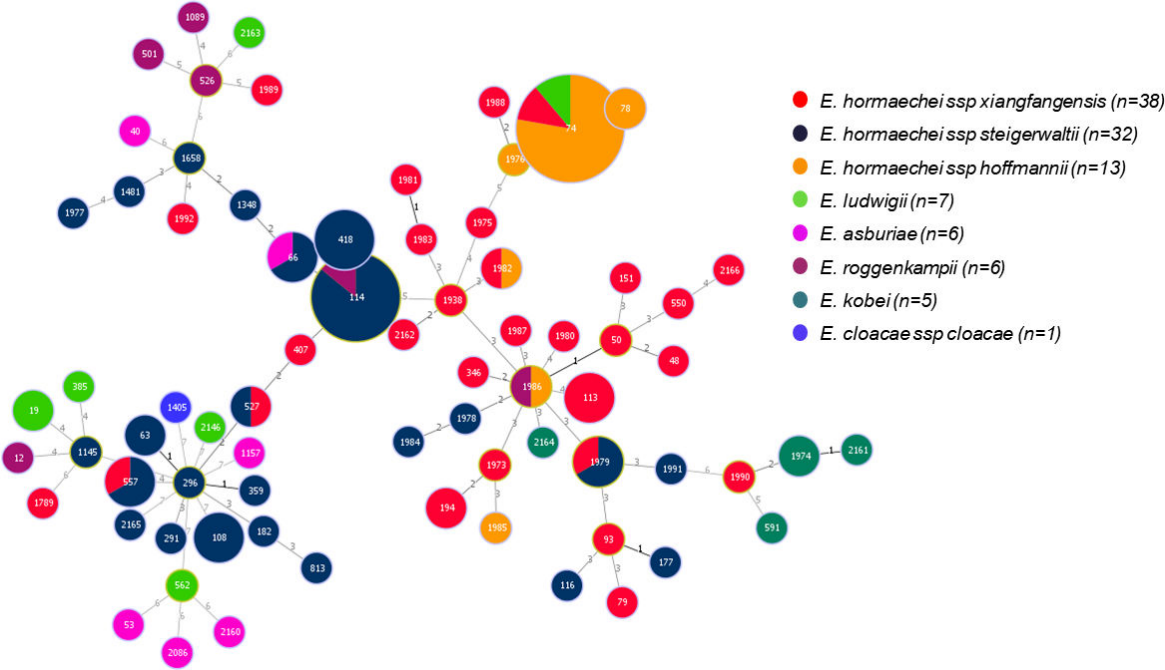
Multilocus sequence typing with minimum spanning tree of 108 clinical isolates of carbapenem-resistant *Enterobacter cloacae* complex. Phylogenetic tree based on neighbor-joining method using *hsp60* gene sequences of 108 clinical isolates and reference strains of *Enterobacter cloacae* complex. Species based on the partial *hsp60* sequences are shown in different colors. Each node within the tree represents a single ST, and the length of branches between each node represents the number of different alleles.

In MLST analysis of 108 CR-ECC isolates, 73 sequence types were identified, including 28 new ones (Fig. 1; Table S4). eBURST analysis, using group definitions of five or more matches, revealed nine groups, including 2 clonal complexes (CCs) and 38 singletons, indicating significant genetic diversity among the isolates (Table S4). The most common ST was ST74 (*n* = 9), with 77.8% identified as *E. hormaechei* subsp. *hoffmannii*, and 88.9% were colistin susceptible. ST74 belongs to CC74, which is known for its high prevalence and association with carbapenem resistance. Consistently, Lee et al. (28, 29) reported that the ST74 clone is linked to the IMP-4 gene, highlighting its role in carbapenemase production and the need for close monitoring due to its clinical implications in antibiotic resistance (28).

The second most common clone was ST114, carrying the *bla*CTX-M-15 gene, with seven isolates, 85.7% of which were identified as *E. hormaechei* subsp. *xiangfangensis*, all colistin susceptible. ST114, along with ST66, is part of CC114, which shows a wide geographical spread and diversification. Both CC74 and CC114 harbor clones associated with significant antibiotic resistance, making them clinically important for their roles in the dissemination of carbapenem and beta-lactam resistance.

### Characteristics of colistin-resistant CR-ECC isolates

The *mcr* gene was isolated in only 27.2% (*n* = 6) of samples, with five *mcr-10* and one *mcr-9* detected in colistin-resistant CR-ECC isolates (*n* = 22). The AST results and MLST of the colistin-resistant CR-ECC isolates are shown in Table 3. These strains were all resistant to colistin but showed relatively good susceptibility to aminoglycosides, tigecycline, fluoroquinolones, and even piperacillin/tazobactam and cefepime.

**TABLE 3:** Antibiotic susceptibility test results and *mcr* gene, carbapenemase status of colistin-resistant CR-ECC strains (*n* = 22)^*a*^^,^

Species	No	*mcr* gene	Carba penemase	CST	TGC	C/T	TZP	AMK	GEN	IPM	MEM	ETP	LVX	CIP	FEP	CTX	ATM	FOF	MLST (ST)
*E. hormaechei* subsp. *hoffmannii*	1	*–^b^*	*–*	4	S	S	S	S	R	S	S	R	R	R	S	R	R	I	74
*E. hormaechei* subsp. *steigerwaltii*	1	*–*	*–*	16	I	R	I	S	S	S	S	R	S	I	R	R	R	R	113
	2	*–*	*–*	8	S	R	S	S	S	S	S	R	S	S	SSD	R	R	S	1,979
	3	*–*	*–*	32	S	R	S	S	S	S	S	R	R	R	S	R	R	I	1,789
	4	*–*	*–*	>32	I	R	R	I	S	S	S	R	R	R	R	R	R	S	1,989
	5	*–*	*–*	8	S	R	R	S	S	S	S	R	S	S	S	R	R	S	79
	6	–	–	>32	S	R	S	I	S	S	S	R	S	S	S	R	R	S	1,990
*E. asburiae*	1	*–*	*–*	>32	S	I	S	S	S	S	S	R	S	S	S	R	R	R	2,160
	2	*–*	*–*	>32	S	S	S	S	S	R	I	R	S	S	S	S	R	S	2,086
	3	*–*	*–*	16	S	R	S	S	S	S	S	R	S	S	S	R	R	I	1,157
	4	*–*	*–*	>32	S	R	S	S	S	S	S	R	S	S	S	R	R	S	40
	5	*–*	*–*	>32	S	R	I	S	S	I	S	R	R	R	S	R	R	S	53
*E. cloacae* subsp. *cloacae*	1	*–*	*–*	8	S	R	I	S	S	S	S	R	S	S	S	R	R	S	1,405
*E. kobei*	1	*mcr-10*	*–*	>32	S	R	S	S	S	S	S	R	S	S	S	R	R	R	1,974
	2	*mcr-10*	*–*	>32	S	R	S	S	S	I	S	R	S	S	S	R	R	S	2,161
	3	*mcr-10*	*–*	>32	S	R	S	S	S	S	S	R	S	S	S	R	R	S	1,974
	4	*–*	*–*	16	S	R	R	S	S	S	S	R	R	R	S	R	R	I	591
*E. ludwigii*	1	*mcr-9*	IMP-1	>128	I	R	R	I	R	I	R	R	R	R	R	R	I	S	2,146
	2	*–*	*–*	>32	I	R	S	S	S	S	S	R	R	R	S	R	R	S	562
*E. roggenkampii*	1	*–*	*–*	>32	S	R	S	S	S	R	I	R	R	R	SSD	R	R	S	1,089
	2	*mcr-10*	*–*	32	S	I	S	S	S	S	S	R	S	S	S	R	R	S	501
	3	*mcr-10*	*–*	>32	S	R	R	S	S	S	S	R	S	S	S	R	R	R	526

^*a*^ MIC was indicated for colistin, and resistance to other antibiotics was indicated (S, susceptible; I, intermediate; and R, resistant).

^*b*^ “–” was indicated for ‘not detected’.

### Clinical characteristics of patients with isolated CR-ECC and risk factors of colistin resistance

Patients with isolated CR-ECC were divided into two groups according to colistin susceptibility, and their clinical characteristics were compared (Table 4). There were no significant differences between the two groups in median age, sex, Charlson comorbidity score, and underlying diseases. CR-ECC were isolated from blood specimens in three (12%) patients in the colistin-non-susceptible group and six (7.2%) in the susceptible group, from rectal swabs in two (8%) vs nine (10.9%), and also from bile, sputum, or urine. The number of cases in which the isolated CR-ECC actually caused clinical infection was 12 (48.0%) vs 31 (37.3%) (*P* = 0.3602). The most common type of infection in both groups was complicated intra-abdominal infection (50% vs 51.6%), followed by skin and soft tissue infection in the colistin-non-susceptible group (25%) and pneumonia/empyema (19.4%) or urinary tract infection (19.4%) in the colistin-susceptible group. The proportion of patients with indwelling catheters was significantly higher in the colistin-susceptible CR-ECC group (32.0% vs 66.2%, *P* = 0.0048), while that of percutaneous biliary drainage catheter was higher in the colistin-non-susceptible group (28.0% vs 10.8%, *P* = 0.0025). There was no difference between the two groups in the detection location (all *P* > 0.05). The treatment success rate was 75.0% vs 87.1%, with no significant difference (*P* = 0.3778). Although they were not significant, the median hospital days were higher in the colistin-non-susceptible CR-ECC group, and the proportion of patients with a history of carbapenem or hospitalization or MDRO detection within 3 months was also higher. No patient in the colistin-non-susceptible group and three (3.6%) patients in the colistin-susceptible group had received colistin administered within 3 months. In the colistin-non-susceptible CR-ECC group, one *mcr-9* and five *mcr-10* genes were detected, compared to six *mcr-9* and no *mcr-10* genes in the colistin-susceptible group. Carbapenemase was detected in one IMP-1 isolate in the colistin-non-susceptible CR-ECC group, whereas six NDM-1, two NDM-5, and one KPC isolate were detected in the colistin-susceptible CR-ECC group.

**TABLE 4:** Baseline characteristics of patients with colistin-non-susceptible carbapenem-resistant *Enterobacter cloacae* complex vs colistin-susceptible carbapenem-resistant *Enterobacter cloacae* complex^*a*^^,^^*e*^

	Colistin-non-susceptible CR-ECC (*n* = 25)	Colistin-susceptible CR-ECC (*n* = 83)	*P*-value
Demographic data			
Age (years)	61.0 (52.5–72.0)	60.0 (49.0–69.0)	0.578
Sex, male (%)	19 (76)	58 (69.9)	0.623
Charlson comorbidity score	6.0 (4.5–6.5)	5.0 (3.0–7.0)	0.325
Underlying conditions			
Solid cancer	19 (79.2)	49 (59.0)	0.159
Solid organ transplantation	3 (12.5)	19 (23.0)	0.395
Hematologic malignancy	2 (8.3)	3 (3.6)	0.327
Microbiological data			
*E. hormaechei* by *hsp60*	9 (37.5)	73 (88.0)	<0.001^*b*^
*E. hormaechei* by VITEK MS	0	17 (20.5)	0.011^*b*^
*mcr* gene or carbapenemase			
*mcr-9*	1 (4)	6 (7.2)	
*mcr-10*	5 (20)	0	
IMP-1	1 (4)	0	
NDM-1	0	6 (7.2)	
NDM-5	0	2 (2.4)	
KPC	0	1 (1.2)	
Clinical data			
Detection site			
Blood	3 (12)	6 (7.2)	
Bile	7 (28)	10 (12.0)	
Sputum	2 (8)	17 (20.5)	
Urine	2 (8)	19 (22.9)	
Peritoneal fluid	3 (12)	14 (16.9)	
Pleural fluid	2 (8)	3 (3.6)	
Skin/pus/wound	4 (16)	3 (3.6)	
Rectal swab	2 (8)	9 (10.9)	
Others^*c*^	0	2 (2.4)	
True infection	12 (48.0)	31 (37.3)	0.360
cIAI	6 (50.0)	16 (51.6)	
SSTI	3 (25.0)	2 (6.4)	
Pneumonia/empyema	2 (16.7)	6 (19.4)	
UTI	1 (8.3)	6 (19.4)	
Vaginitis	0	1 (3.2)	
Treatment success^*d*^	9 (75.0)	27 (87.1)	0.378
Hospital days	10 (3.5–31.0)	6 (1.0–19.0)	0.102
Indwelling catheter	8 (32.0)	55 (66.2)	0.005^*b*^
PTBD	7 (28.0)	9 (10.8)	0.003^*b*^
CVC	4 (16.0)	15 (18.1)	>0.999
Foley	3 (12.0)	22 (26.5)	0.179
Chest tube	3 (12.0)	10 (12.0)	>0.999
Location of detection			
General ward	18 (72.0)	52 (62.7)	0.478
ICU	5 (20.0)	19 (22.9)	>0.999
ER/outpatient	1 (4.0)	11 (13.3)	0.288
Carbapenem within 3 months	5 (20.0)	15 (18.1)	0.777
Colistin within 3 months	0	3 (3.6)	>0.999
MDRO within 3 months	6 (24.0)	13 (15.7)	0.373
Hospitalization within 3 months	20 (80.0)	54 (65.1)	0.220
Hemodialysis	5 (20.0)	24 (28.9)	0.449

^*a*^ Data are expressed as the number (%) of patients or median (IQR) values.

^*b*^ Statistically significant difference was noticed.

^*c*^ One genital tract and one unspecified tissue.

^*d*^ Only clinically significant infections were analyzed.

^*e*^ cIAI, complicated intraabdominal infection; SSTI, skin and soft tissue infection; UTI, urinary tract infection; PTBD, percutaneous biliary drainage catheter; CVC, central venous catheter; ICU, intensive care unit; ER, emergency room; and MDRO, multidrug resistant organisms

Logistic regression analysis was performed to find risk factors for colistin resistance among CR-ECC (Table 5). In both univariable (HR 0.068, CI 0.023–0.0200, *P* < 0.001) and multivariable analyses (HR 0.089, CI 0.030–0.261, *P* < 0.001), the only protective factor for colistin resistance was *E. hormaechei* identified with the *hsp60* method.

**TABLE 5:** Risk factors for colistin-resistant carbapenem-resistant *Enterobacter cloacae* complex

Variables	Univariable analysis		Multivariable analysis	
	OR (95% CI)	*P*-value	Adjusted OR (95% CI)	*P*-value
Age over 60 years	0.717 (0.289–1.778)	0.472		
Male	1.365 (0.487–3.826)	0.554		
*E. hormaechei* in VITEK MS	<0.001	0.998	<0.001	0.998
*E. hormaechei* in *hsp60*	0.068 (0.023–0.200)	<0.001	0.089 (0.030–0.261)	<0.001
Clinical infection	1.548 (0.628–3.816)	0.342		
Drain tube	1.309 (0.489–3.504)	0.592		
Carbapenem within 3 months	1.133 (0.367–3.502)	0.828		
Colistin within 3 months	<0.001	0.999		
MDRO detection within 3 months	1.700 (0.570–5.068)	0.341		
Hospitalization within 3 months	2.148 (0.730–6.318)	0.165		
Hemodialysis	0.823 (0.088–7.718)	0.823		

When analyzing only the 43 isolates that caused true infection, *E. cloacae* complex was most common at 51.2%, followed by *E. cloacae* at 39.5% and *E. hormaechei* at 9.3% in VITEK MS. In the *hsp60* gene analysis, *E. hormaechei* subsp. *xiangfangensis* was the most common at 41.9%, followed by *E. hormaechei* subsp. *steigerwaltii* (23.2%), *E. asburiae* (14%), and *E. hormaechei* spp. *hoffmannii* (9.3%). There were no significant differences in baseline characteristics between the true pathogen group (*n* = 43) and the colonizer group (*n* = 65) (all *P* > 0.05). Logistic regression also did not identify any risk factors with significant association with true clinical infection (all *P* > 0.05).

## DISCUSSION

Species identification of the isolates showed that *E. hormaechei* was the most commonly isolated strain among the seven ECC species. The proportion of colistin-non-susceptible CR-ECC was high at 23.2%. The colistin resistance rates of non-*E*. *hormaechei* were higher than that of *E. hormaechei* (28.6%–100% vs 0%–18.8%). In a recent study on ECC conducted in Japan, all *E. hormaechei* were colistin susceptible, consistent with the results of the present study (30). However, there are few extant works of research about colistin resistance in *E. hormaechei*, and those that exist have been conducted relatively recently.

The problem with the clinical treatment of emerging CRE infections is that there are few appropriate antibiotic treatment options. Currently, colistin is administered empirically in cases of carbapenem resistance and when there are no other antibiotic options. However, the VITEK used clinically does not report colistin susceptibility; even if it is reported, the reliability is low, and there is always a possibility of treatment failure. In a previous study (31), GNB including Enterobacterales were tested for colistin resistance using the VITEK2 system and BMD, and the proportion of very major errors (VMEs) of reported colistin resistance in BMD but sensitivity in VITEK2 reached 10%. In particular, when only *E. cloacae* was analyzed, 35% of strains were falsely reported as sensitive in VITEK2. In another study, the rates of essential agreement of colistin testing for VITEK2 and BMD were 93.4%, but the categorical agreement was less than 90% and the VME rate was 36%. This may lead to treatment failure in the current situation where colistin resistance in ECC (either carbapenem susceptible or non-susceptible) has been reported to be as high as 39.9% (17), especially in critically ill patients. However, in the present study, all isolates initially identified as *E. hormaechei* by VITEK MS were colistin sensitive. Therefore, once identified as *E. hormaechei* by VITEK MS, these isolates might be considered safely treatable with colistin, despite the differences in identification results between the VITEK MS and hsp60 sequencing method for all ECCs. This was confirmed in logistic regression conducted to predict risk factors for colistin resistance. Furthermore, in 2020, recognizing the continued reliance on colistin in regions where new drugs are not universally available or effective, the CLSI revised the colistin breakpoint (32). Therefore, in hospitals like those in Korea, where newer drugs are not widely available, routine colistin testing of ECC can help guide antibiotic choices. However, consultation with infectious disease specialists is essential for the accurate interpretation of reported results and their application to clinical treatment.

In MLST analysis, ST74 was the predominant clonal lineage with increased epidemic potential based on previous *E. cloacae* clonality studies (28, 33, 34). *E. cloacae* ST74 had higher carbapenem MICs than other isolates, similar to the results of previous studies, and was assumed to confer with the spread of the resistance to carbapenems (28, 34). The second most identified ST114 was described by Girlich et al. (35) as the most prevalent in a worldwide collection of *bla*CTX-M-15-producing ECC of clinical origin and has been reported in a hospital outbreak (36).

Another notable point in this study is that susceptibility to several antibiotics was relatively preserved in CR-ECC isolates. The classification as CR-ECC was because of resistance to ertapenem, but there were many isolates susceptible to other carbapenems (about 90%). If these isolates do not have a carbapenemase gene, extended-infusion meropenem or imipenem-cilastatin could be administered according to the guidelines. Resistance to aminoglycosides was found in approximately 10% of isolates, susceptibility to piperacillin/tazobactam was preserved in 57.4% of cases, and 71.3% of cases were susceptible to tigecycline. These well-preserved antibiotic susceptibilities are one of the reasons why there was no difference in prognosis according to colistin susceptibility. Therefore, it is possible to administer other antibiotics with preserved susceptibility other than colistin even in CR-ECC.

In this study, the *mcr* gene was detected in only 27.2% of colistin-resistant isolates. This is consistent with the results of a previous study showing that CR-ECC had the highest *mcr* gene detection rate among CRE isolates in South Korea at 20.5% (27), and with the results of another study showing a detection rate of 35.9% (37). Additionally, one *mcr-9* and five *mcr-10* genes were found in the colistin-non-susceptible group and six *mcr-9* and no *mcr-10* genes were found in the colistin-susceptible group. This is consistent with previous data that most *mcr-9* genes were detected in colistin-susceptible *Enterobacter* subsp., and *mcr-10* genes were associated with colistin-non-susceptible *Enterobacter* subsp. (38, 39). Previous studies have shown that *mcr-9/10* genes do not directly confer colistin resistance but are also involved in other mechanisms such as bacterial immunity to the host immune system or conferring resistance to other cationic antimicrobial peptides (40). These results also suggest that *mcr-10* genes may show a stronger association with colistin resistance than *mcr-9* genes and further studies are needed.

The proportion of isolates producing carbapenemase was 8.3% in this study, which was lower than reported data in South Korea. According to the CPE infection report from the KDCA (6, 41), CPE accounted for 63.4% of CRE infection (*n* = 14,769) in 2021 and 71.0% (*n* = 21,695) of CRE infections (*n* = 30,548) in 2022. This discrepancy can be attributed to the lower CPE prevalence of the study center compared to other hospitals. Among CREs isolated from our center from January 2021 to June 2023, CPE was found in 38.5%, with the most common type being KPC, followed by NDM, OXA, and IMP (J. Yang, unpublished data). Also, there may be differences because the data published by the KDCA included all Enterobacteriaceae, while the results of the present study were limited to ECC. The results of this study should be interpreted considering the inclusion of only isolates from a specific center in South Korea and a relatively small sample size.

In this study, we discovered an *E. ludwigii* isolate co-harboring *mcr-9.1* and multicopies of *bla_IMP-1_* gene and reported its whole genome sequence and characteristics (42). The isolate had a very high colistin MIC greater than 128, and the emergence of such an extensively drug-resistant isolate could be a significant threat within compatible Enterobacteriaceae.

One of the limitations of this study is that analysis of heteroresistance to colistin in CRE was not conducted. A previous study showed a heteroresistance rate of *Enterobacter* spp. of 20%, which is a much higher rate than those of *Klebsiella* subsp. (8.4%) and *Escherichia* subsp. (2.1%) (43). Therefore, additional studies on colistin heteroresistance in CR-ECC should be conducted. Another limitation is that this study only targeted strains collected from a single center, and it may be difficult to generalize the results.

### Conclusions

Although colistin resistance of CR-ECC is relatively high, colistin could be administered safely to treat infections with *E. hormaechei*. It is recommended to select an alternative option with well-preserved antibiotic susceptibility in addition to colistin, and it is imperative to maintain ongoing surveillance and to further research on CR-ECC.

## SUPPLEMENTAL MATERIAL

Supplemental tables spectrum.01604-24-s0001.docx Tables S1 to S4.

ASM does not own the copyrights to Supplemental Material that may be linked to, or accessed through, an article. The authors have granted ASM a non-exclusive, world-wide license to publish the Supplemental Material files. Please contact the corresponding author directly for reuse.
