# Supplementary material for: Clinical and microbiological analyses of colistin-resistant strains among carbapenem-resistant Enterobacter cloacae complex clinical isolates
Source: Microbiol Spectr. 2024 Dec 31;13(2):e01604-24. doi: 10.1128/spectrum.01604-24 (PMC11792525; doi:10.1128/spectrum.01604-24)
Supplement: Supplemental tables — Tables S1 to S4. [file spectrum.01604-24-s0001.docx]

Supplementary Table 1. Reference strains of *hsp60* sequences used for species identification of the *Enterobacter cloacae* complex (ECC) isolates

| **Reference species or subspecies** | **Strain Number** |
| --- | --- |
| *E. asburiae* | ATCC35953 |
| *E. kobei* | J68 |
| *E. ludwigii* | 11894 |
| *E. ludwigii* | CEB04 |
| *E. roggenkampii* | DSM 16690 |
| *E. cloacae* subsp. *cloacae* | ATCC 13047 |
| *E. hormaechei* subsp. *hoffmannii* | DSM 14563 |
| *E. hormaechei* subsp. *xiangfangensis* | 34399 |
| *E. hormaechei* subsp. *xiangfangensis* | LMG 27195 |
| *E. hormaechei* subsp. *hormaechei* | EN 449 |
| *E. hormaechei* subsp. *steigerwaltii* | ME-1 |

Supplementary Table 2. The similarity of partial *hsp60* sequences between reference strains and clinical isolates of subspecies of *E. hormaechei*.

| **Subspecies** | **subsp. *hoffmannii* DSM14563** | **subsp. *hormaechei* EN449** | **subsp. *steigerwaltii* ME-1** | **subsp. *xiangfangensis* LMG27195** |
| --- | --- | --- | --- | --- |
| subsp. *hoffmannii* | 99.56–100 | 93.83–94.27 | 94.27–94.71 | 93.83–94.27 |
| subsp. *steigerwaltii* | 94.27–94.71 | 98.68–99.12 | 99.56–100 | 98.24–98.68 |
| subsp. *xiangfangensis* | 93.83–94.27 | 97.36–98.24 | 98.24–99.12 | 98.68–100 |

| Species | Carba-  penemase | CST | TGC | C/T | TZP | AMK | GEN | IPM | MEM | ETP | LVX | CIP | FEP | CTX | ATM | FOF |
| --- | --- | --- | --- | --- | --- | --- | --- | --- | --- | --- | --- | --- | --- | --- | --- | --- |
| *E. ludwigii* | IMP-1 | R | I | R | R | I | R | I | R | R | R | R | R | R | I | S |
|  | NDM-1 | S | I | R | R | S | S | R | R | R | R | R | R | R | R | I |
|  | NDM-5 | S | S | R | R | S | S | R | R | R | S | S | R | R | S | R |
| *E. hormaechei*  subsp*. hoffmannii* | NDM-1 | S | S | R | R | S | R | R | R | R | R | R | R | R | R | S |
|  | NDM-1 | S | S | R | S | S | S | S | S | R | R | R | R | R | R | I |
|  | NDM-5 | S | R | R | R | S | S | R | R | R | R | R | R | R | R | S |
| *E. hormaechei*  subsp*. xiangfangensis* | NDM-1 | S | I | R | R | S | S | R | R | R | R | R | R | R | I | S |
|  | NDM-1, KPC-2 | S | I | R | R | S | S | R | R | R | R | R | R | R | R | S |
| *E. hormaechei*  subsp*. steigerwaltii* | NDM-1 | S | S | R | R | S | S | R | R | R | S | S | R | R | S | S |

Supplementary Table 3. Antibiotic susceptibility results of carbapenemase-producing carbapenem-resistant *Enterobacter cloacae* complex isolates (n=9)

MIC was indicated for colistin, and resistance to other antibiotics was indicated. (S, susceptible; I, intermediate; R, resistant)

Supplementary Table 4. Clonal diversity among clinical isolates of *Enterobacter cloacae* Complex (ECC) in Korea

| **Clonal status ^a^**  **(no. of isolate)** | **ST ^b^** | **No. of isolate** | ***dnaA*** | ***fusA*** | ***gyrB*** | ***leuS*** | ***pyrG*** | ***rplB*** | ***rpoB*** | **Most prevalent species within group (n, %)** |
| --- | --- | --- | --- | --- | --- | --- | --- | --- | --- | --- |
| **Group 1**  **CC74 (11)** | 74 | 9 | 8 | 33 | 6 | 9 | 9 | 6 | 8 | *E. hormaechei* subsp *hoffmannii* (9, 81.8%) |
|  | 78 | 2 | 8 | 9 | 6 | 9 | 9 | 6 | 8 |  |
| **Group 2**  **CC114 (23)** | 63 | 2 | 49 | 20 | 19 | 45 | 45 | 4 | 32 | *E. hormaechei* subsp *xiangfangensis* (17, 73.9%) |
|  | 66 | 3 | 52 | 21 | 20 | 44 | 45 | 4 | 6 |  |
|  | 114 | 7 | 53 | 35 | 20 | 44 | 45 | 4 | 6 |  |
|  | 418 | 4 | 53 | 35 | 154 | 44 | 45 | 4 | 6 |  |
|  | 296 | 1 | 49 | 20 | 19 | 65 | 45 | 4 | 32 |  |
|  | 527 | 2 | 53 | 20 | 19 | 178 | 45 | 4 | 32 |  |
|  | 359 | 1 | 125 | 20 | 19 | 65 | 45 | 4 | 32 |  |
|  | 407 | 1 | 53 | 35 | 19 | 44 | 45 | 4 | 32 |  |
|  | 1348 | 1 | 46 | 21 | 19 | 44 | 45 | 4 | 6 |  |
|  | 1658 | 1 | 46 | 21 | 19 | 44 | 45 | 12 | 32 |  |
| **Group 3 (7)** | **1986** | 2 | 4 | 6 | 4 | 6 | 37 | 4 | 25 | *E. hormaechei* subsp *steigerwaltii* (3, 42.9%) |
|  | 50 | 1 | 4 | 4 | 4 | 6 | 37 | 4 | 25 |  |
|  | 48 | 1 | 4 | 4 | 4 | 39 | 41 | 4 | 25 |  |
|  | 346 | 1 | 4 | 89 | 4 | 6 | 37 | 4 | 78 |  |
|  | **1978** | 1 | 4 | 6 | 4 | 40 | 39 | 4 | 25 |  |
|  | **1984** | 1 | 4 | 6 | 13 | 4 | 39 | 4 | 25 |  |
| **Group 4 (4)** | **1974** | 2 | 63 | 11 | 66 | 68 | 3 | 16 | 3 | *E. kobei* (3, 75%) |
|  | **1990** | 1 | 66 | 3 | 66 | 68 | 3 | 16 | 3 |  |
|  | **2161** | 1 | 63 | 11 | 66 | 68 | 514 | 16 | 3 |  |
| **Group 5 (3)** | 194 | 2 | 11 | 6 | 4 | 13 | 39 | 4 | 9 | *E. hormaechei* subsp *hoffmannii* (2, 66.7%) |
|  | **1973** | 1 | 62 | 6 | 4 | 6 | 39 | 4 | 9 |  |
| **Group 6 (2)** | **1976** | 1 | 59 | 33 | 6 | 9 | 106 | 6 | 6 | *E. hormaechei* subsp *hoffmannii* (1, 50%) *E. hormaechei* subsp *steigerwaltii* (1, 50%) |
|  | **1988** | 1 | 59 | 40 | 6 | 9 | 67 | 6 | 6 |  |
| **Group 7 (2)** | 1938 | 1 | 4 | 6 | 14 | 6 | 39 | 4 | 6 | *E. hormaechei* subsp *steigerwaltii* (2, 100%) |
|  | **2162** | 1 | 4 | 6 | 568 | 6 | 39 | 4 | 39 |  |
| **Group 8 (2)** | **1981** | 1 | 58 | 6 | 257 | 4 | 39 | 4 | 151 | *E. hormaechei* subsp *steigerwaltii* (2, 100%) |
|  | **1983** | 1 | 58 | 6 | 14 | 4 | 39 | 4 | 151 |  |
| **Group 9 (2)** | 93 | 1 | 9 | 4 | 14 | 61 | 37 | 4 | 9 | *E. hormaechei* subsp *steigerwaltii* (1, 50%) *E. hormaechei* subsp *xiangfangensis* (1, 50%) |
|  | 177 | 1 | 9 | 4 | 14 | 61 | 89 | 4 | 9 |  |
| **Singleton ^c^ (52)** | 108 | 3 | 68 | 8 | 75 | 63 | 65 | 34 | 35 | ***E. hormaehaei*** (34)  *E. hormaechei* subsp *steigerwaltii* (16)  *E. hormaechei* subsp *xiangfangensis* (13)  *E. hormaechei* subsp *hormaechei* (3)  *E. hormaechei* subsp *hoffmannii* (2)  ***Non-E. hormaechei*** (18)  *E. asburiae* (5)  *E. ludwigii* (6)  *E. roggenkampii* (4)  *E. kobei* (2)  *E. cloacae* subsp *cloacae* (1) |
|  | 113 | 3 | 4 | 22 | 68 | 69 | 37 | 4 | 24 |  |
|  | 557 | 3 | 186 | 20 | 9 | 95 | 45 | 12 | 32 |  |
|  | **1979** | 3 | 9 | 6 | 15 | 6 | 37 | 4 | 9 |  |
|  | 19 | 2 | 18 | 2 | 41 | 22 | 51 | 2 | 13 |  |
|  | 1982 | 2 | 360 | 6 | 391 | 6 | 39 | 74 | 6 |  |
|  | 12 | 1 | 13 | 2 | 45 | 24 | 52 | 2 | 14 |  |
|  | 40 | 1 | 36 | 26 | 36 | 49 | 50 | 12 | 20 |  |
|  | 53 | 1 | 40 | 17 | 39 | 15 | 46 | 11 | 10 |  |
|  | 79 | 1 | 9 | 22 | 14 | 6 | 39 | 4 | 9 |  |
|  | 116 | 1 | 9 | 4 | 14 | 6 | 11 | 4 | 6 |  |
|  | 151 | 1 | 4 | 4 | 4 | 77 | 11 | 4 | 39 |  |
|  | 291 | 1 | 52 | 20 | 20 | 118 | 45 | 4 | 32 |  |
|  | 385 | 1 | 123 | 2 | 136 | 136 | 2 | 2 | 14 |  |
|  | 501 | 1 | 37 | 25 | 49 | 30 | 49 | 82 | 20 |  |
|  | 550 | 1 | 179 | 4 | 4 | 6 | 112 | 4 | 6 |  |
|  | 562 | 1 | 22 | 15 | 102 | 104 | 101 | 11 | 71 |  |
|  | 591 | 1 | 3 | 3 | 110 | 232 | 19 | 16 | 17 |  |
|  | 813 | 1 | 74 | 20 | 20 | 44 | 99 | 24 | 32 |  |
|  | 1089 | 1 | 81 | 199 | 92 | 92 | 49 | 12 | 26 |  |
|  | 1157 | 1 | 267 | 211 | 352 | 399 | 319 | 85 | 144 |  |
|  | 1145 | 1 | 15 | 2 | 229 | 386 | 51 | 2 | 14 |  |
|  | 1405 | 1 | 78 | 107 | 61 | 463 | 83 | 22 | 248 |  |
|  | 1481 | 1 | 46 | 20 | 74 | 44 | 45 | 12 | 244 |  |
|  | 1789 | 1 | 216 | 158 | 263 | 133 | 56 | 105 | 14 |  |
|  | **1975** | 1 | 9 | 6 | 15 | 13 | 39 | 6 | 6 |  |
|  | **1977** | 1 | 74 | 20 | 74 | 387 | 308 | 12 | 32 |  |
|  | **1980** | 1 | 51 | 6 | 4 | 6 | 68 | 30 | 36 |  |
|  | **1985** | 1 | 51 | 22 | 4 | 6 | 39 | 4 | 186 |  |
|  | **1987** | 1 | 4 | 6 | 4 | 425 | 4 | 4 | 4 |  |
|  | **1989** | 1 | 343 | 38 | 67 | 48 | 331 | 12 | 26 |  |
|  | **1991** | 1 | 66 | 6 | 15 | 40 | 76 | 4 | 9 |  |
|  | **1992** | 1 | 10 | 21 | 17 | 40 | 134 | 12 | 32 |  |
|  | 2086 | 1 | 22 | 225 | 449 | 678 | 411 | 65 | 366 |  |
|  | **2146** | 1 | 280 | 178 | 299 | 692 | 274 | 106 | 156 |  |
|  | **2160** | 1 | 558 | 195 | 293 | 695 | 21 | 11 | 58 |  |
|  | **2163** | 1 | 261 | 26 | 71 | 696 | 169 | 224 | 26 |  |
|  | **2164** | 1 | 4 | 6 | 15 | 697 | 37 | 4 | 6 |  |
|  | **2165** | 1 | 559 | 16 | 25 | 53 | 127 | 9 | 15 |  |
|  | 182 | 1 | 49 | 20 | 19 | 44 | 90 | 24 | 32 |  |
|  | 526 | 1 | 37 | 27 | 35 | 126 | 49 | 12 | 26 |  |
|  | **2166** | 1 | 179 | 4 | 569 | 305 | 515 | 274 | 6 |  |

^a^ Clonal status of an ST is defined as membership of an eBURST group (an apparent clonal complex (CC), a group defined as five or more matches for which no central genotype distinguished in the database) or being a singleton.

^b^ STs identified in the study; numbers in bold indicate new STs.

^c^ Singletons were classified as *E. hormaechei* subspecies and non-*E. hormaechei* subspecies, showing a significant difference in colistin resistance (66.7% vs 14.7%, P = 0.0023).

MLST analysis of 108 CR-ECC isolates identified 73 sequence types (STs), including 28 new ones. eBURST analysis, with group definitions of 5 or more matches, revealed 9 groups, 2 clonal complexes (CCs), and 38 singletons, indicating significant genetic diversity among the isolates (Table S4).
